# Effects of swab pool size and transport medium on the detection and isolation of avian influenza viruses in ostriches

**DOI:** 10.1186/s12917-022-03150-6

**Published:** 2022-01-18

**Authors:** Reneé Pieterse, Christine Strydom, Celia Abolnik

**Affiliations:** 1 Provincial Veterinary Laboratory, Western Cape Department of Agriculture, Helshoogte Road, Stellenbosch, 7600 South Africa; 2 Department of Veterinary Tropical Diseases, University of Pretoria Faculty of Veterinary Science, Old Soutpan Road, Onderstepoort, 0110 South Africa; 3 NOSA Testing (Pty) Ltd, 248 Jean Avenue, Centurion, Lyttleton 0140 South Africa; 4 SMT Veterinary Laboratory, Irene, Pretoria, 0178 South Africa; 5 Department of Production Animal Studies, University of Pretoria Faculty of Veterinary Science, Old Soutpan Road, Onderstepoort, 0110 South Africa

**Keywords:** Influenza a virus, Ostrich, Viral transport medium, Tracheal swab pooling

## Abstract

**Background:**

Rigorous testing is a prerequisite to prove freedom of notifiable influenza A virus infections in commercially farmed ostriches, as is the isolation and identification of circulating strains. Pooling 5 ostrich tracheal swabs in a 50 % v/v phosphate-buffered saline (PBS): glycerol transport medium (without antibiotics) is the current standard practice to increase reverse transcription real time PCR (RT-rtPCR) testing throughput and simultaneously reduce the test costs. In this study we investigated whether doubling ostrich tracheal swabs to 10 per pool would affect the sensitivity of detection of H5N8 high pathogenicity avian influenza virus (HPAIV) and H7N1 low pathogenicity avian influenza virus (LPAIV) by quantitative RT-rtPCR, and we also compared the effect of a protein-rich, brain heart infusion broth (BHI) virus transport media containing broad spectrum antimicrobials (VTM) on the efficacy of isolating the H5N8 and H7N1 viruses from ostrich tracheas, since the historical isolation success rate from these birds has been poor.

**Results:**

Increasing the ostrich swabs from 5 to 10 per pool in 3 mls of transport medium had no detrimental effect on the sensitivity of the RT-rtPCR assay in detecting H5N8 HPAIV or H7N1 LPAIV; and doubling of the swab pool size even seemed to improve the sensitivity of virus detection at levels that were statistically significant (*p* less than or equal to 0.05) in medium and low doses of spiked H5N8 HPAIV and at high levels of spiked H7N1 LPAIV. On virus isolation, more samples were positive when swabs were stored in a protein-rich viral transport medium supplemented with antimicrobials in PBS: glycerol (10/18 vs. 7/18 for H5N8 HPAI); although the differences were not statistically significant, overall higher virus titres were detected (10^6.7^ – 10^3.0^ vs. 10^6.6^ - 10^3.1^ EID_50_ for H5N8 HPAIV and 10^5.5^ - 10^1.4^ vs. 10^5.1^ – 10^1.3^ EID_50_ for H7N1 LPAIV); and fewer passages were required with less filtration for both H5N8 HPAI and H7N1 LPAI strains.

**Conclusion:**

Ostrich tracheal swab pool size could be increased from 5 to 10 in 3mls of VTM with no loss in sensitivity of the RT-rtPCR assay in detecting HPAI or LPAI viruses, and HPAI virus could be isolated from a greater proportion of swabs stored in VTM compared to PBS: glycerol without antibiotics.

## Background

Ostriches (*Struthio camelus*) are highly versatile production animals raised for their low-cholesterol, lean red meat; fine leather, and feathers used in the fashion industry. Namibia, Australia, Botswana, France, Indonesia, and some other territories like China, the USA, and the Middle East to a lesser extent raise ostriches commercially, but South Africa remains the lead export producer with 75% of the global market share, and the European Union (EU) as the main market [1]. The number of slaughter birds in South Africa has been on a constant decline from 250,000 per annum in 2011 to < 150,000 in 2020. Drought in the region has played a role, but the most significant driver of the decline is virus identification-related long delays to regain freedom of avian influenza (AI) infection status. Ostrich producers aiming to export ostrich meat must be registered with the competent authority and comply with strict bio-security requirements, amongst other measures. The fresh meat of birds originating from a holding that was exposed to notifiable avian influenza (i.e., viruses of the H5 or H7 subtypes) within 6 months prior to slaughter may not be exported; therefore, routine screening of ostriches to prove freedom of infection from influenza A virus (IAV) infection is central to maintaining export status [2].

The extensive nature of the farming system predisposes ostriches to frequent contact with wild birds. Wild waterfowl species are the natural reservoirs of all low pathogenicity influenza A viruses (LPAIV) as well as clade 2.3.4.4 H5Nx high pathogenicity influenza A viruses (HPAIV) [3, 4], thus any IAVs excreted in sufficient titres in their faeces and oral secretions into a shared environment may be ingested or inhaled by the ostriches. Atypically for a terrestrial species, ostriches normally show no clinical signs with LPAIV or HPAIV infection [4, 5] yet like gallinaceous birds, ostriches can facilitate mutation of LPAIV to HPAIV after a period of intra-host virus circulation. The emergence of H5N2 HPAIV in ostriches caused three unrelated epidemics in 2004, 2006 and 2011 in South Africa’s Eastern and/or Western Cape provinces [5], and ostriches also contracted clade 2.3.4.4B H5N8 HPAIV strains introduced by migratory waterfowl in the 2017–2018 epidemics [4, 5]. Regardless of the implications for the export market, early detection and preventing notifiable IAVs from circulating in ostriches is very important, as any spill-over to the mainstream poultry industry could threaten regional food security or even human health.

In South Africa, serological surveillance for IAV infection of all registered ostrich farms is compulsory every 6 months. When H5 or H7-specific antibodies are detected, immediate disease investigation and control measures are implemented, including intensified sampling for serological testing and agent detection using real-time reverse transcription polymerase chain reaction (RT-rtPCR). Repeated sampling events must be done to detect the presence or absence of AI-specific antibodies and the virus antigen at a > 5% prevalence with 95% confidence in each epidemiological group. Any RT-rtPCR positive results must be tested further to identify the IAV subtype and pathotype [2, 4]. Where the presence of HPAI is confirmed, strict quarantine and movement restrictions come into effect and suspension of export from the infected zones, province and/ or the country. Only when the infected zone or compartment is free from infection for at least 6 months from the date of the last infected farm or compartment is the outbreak declared over to the World Animal Health Organization (OIE), and exports can resume [2].

Ostrich tracheal swabs contain higher viral titres than cloacal swabs [6], therefore the standard practice in South Africa is to collect tracheal swabs, pooling a maximum of five into a single tube containing 50% v/v phosphate-buffered saline (PBS): glycerol medium that, notably, does not contain antimicrobials. Virus isolation is the gold standard for agent detection [7] but is not performed routinely with RT-rtPCR-positive ostrich samples for reasons such as cost and the limited supply of Specific Antigen Negative or Specific Pathogen Free (SPF) embryonated chicken eggs (ECEs). Furthermore, the success rates where egg isolations were attempted on ostrich tracheal swab fluids with RT-rtPCR cycle threshold values < 30 have been inexplicably poor, certainly lower than that for chickens for which the same sampling methods are used [4]. For example, from March to November 2019 a national laboratory tested 977 ostrich swab pools of which 109 (11.2 %) were IAV positive by RT-rtPCR. None were typed as H5/H7 positive on RT-rtPCR, and only one virus was isolated and later identified as the H11N1 subtype (A. Olivier personal communication; C. Abolnik, unpublished diagnostic case reports). During the 2011 H5N2 HPAI outbreak, only three viruses were isolated from swab pools of twenty ostrich farms with multiple RT-rtPCR-positive pools each [8] and in the 2017–2018 H5N8 HPAI outbreaks, 38 ostrich tracheal swab pools were H5N8 HPAIV RT-rtPCR-positive, but only two viruses were isolated whereas 39 viruses were isolated from the oropharyngeal and cloacal swabs or tissue samples of other avian species [4].

To reduce testing costs but still meet statistical sampling requirements to detect the presence of IAV infection, the OIE and other sources recommend that up to eleven swabs from chickens or turkeys may be pooled without a loss in sensitivity of the RT-rtPCR assay [7, 9, 10], but since there may be differences between shedding patterns for different hosts [11], the pooling of more than 5 ostrich swabs is not permitted by the national veterinary authority. Therefore, in this study we used spiking experiments to evaluate whether increasing ostrich tracheal swabs from five to ten per pool would affect the sensitivity of H5N8 HPAIV or H7N1 LPAIV detection by quantitative RT-rtPCR. We also compared the efficacy of H5N8 HPAIV and H7N1 LPAIV isolation from ostrich tracheal swabs in the standard PBS: glycerol medium without antimicrobials to a protein-based viral transport medium containing antimicrobials, since our recent study [12] identified specific bacteria in ostrich tracheal swabs that directly affect the viability of IAVs.

## Results

### Effect of swab pool size on the sensitivity of the detection of IAV by quantitative RT-rtPCR

Specific RNA was detected by quantitative RT-rtPCR in all six replicates for the 5- and 10- swab pools for samples spiked with low, medium and high H5N8 HPAIV concentrations (Table 1). There was no statistically significant difference (*p* ≤ 0.05) between the mean Ct values obtained for the 5- and 10-swab pools for H5N8 HPAIV at the highest virus concentration (virus titres of 10^6.0^ vs. 10^6.1^ EID_50_/ ml), but slightly higher viral titres were detected in the 10-swab pools spiked with the medium dose (10^3.9^ vs. 10^4.2^ EID_50_/ ml) and the low dose (10^2.2^ vs. 10^2.8^ EID_50_/ ml) that were statistically significant (*p* ≤ 0.05).

**Table 1:** H5N8 HPAI virus detected by RT-rtPCR in 5 vs. 10 swabs/ pool stored in VTM^a^

Spiking dose	5 swabs/ pool				10 swabs/ pool				***P*** (p ≤ 0.05)
	Mean virus titre in EID_**50**_ equivalents^b^/ ml	Mean Ct value	SD	Positives	Mean virus titre in EID_**50**_ equivalents^b^/ ml	Mean Ct value	SD	Positives	
High	10^6.0^	22.9	0.12	6/6	10^6.1^	22.4	0.48	6/6	0.0556
Medium	10^3.9^	30.3	0.29	6/6	10^4.2^	29.4	0.66	6/6	0.0293^*^
Low	10^2.2^	36.5	0.25	6/6	10^2.8^	34.3	0.46	6/6	0.0006^*^

*Ct* cycle threshold, *SD* Standard deviation

^a^Viral Transport Medium- brain-heart infusion broth (pH 7.2), 10% (v/v) glycerol and antimicrobials per litre: 100 mg doxycycline, 100 mg enrofloxacin, 1000 mg penicillin-streptomycin, 5 mg Amphotericin B

^b^Egg Infectious Dose _50_ equivalents as determined by RT-rtPCR

^*^Statistically significant

Similarly, H7N1 LPAIV RNA was detected in all six replicates for the 5- and 10- swab pools for samples spiked with varying virus concentrations (Table 2) however a statistically significant difference (*p* ≤ 0.05) in the Ct values was only detected in the highest spiking concentrations with titres of 10^5.1^ vs. 10^5.5^ EID_50_/ ml. The differences in titres of 10^2.9^ vs. 10^3.1^ EID_50_/ ml for the medium concentration and 10^1.3^ vs. 10^1.4^ EID_50_/ ml for the low concentration were not statistically significant (*p* > 0.05), even though the quantities of H7N1 LPAIV detected in the 10-swab pools were slightly higher than the 5-swab pool samples, similar to H5N8 HPAIV. Increasing the swab pool size from 5 to 10 thus had no detrimental effect on the sensitivity of the RT-rtPCR assay in detecting H5N8 HPAIV or H7N1 LPAIV; doubling the swab pool size even seemed to be beneficial overall.

**Table 2:** H7N1 LPAI virus detected by RT-rtPCR in 5 vs. 10 swabs/ pool stored in VTM^a^

Spiking dose	5 swabs/ pool				10 swabs/ pool				***P*** (*p* ≤ 0.05)
	Mean virus titre in EID_**50**_ equivalentsViral Transport Medium- brain-heart infusion ^b^/ ml	Mean Ct value	SD	Positives	Mean virus titre in EID_**50**_ equivalents^b^/ ml	Mean Ct value	SD	Positives	
High	10^5.1^	24.2	0.24	6/6	10^5.5^	23.1	0.29	6/6	0.0011^*^
Medium	10^2.9^	31.4	0.84	6/6	10^3.1^	30.8	1.28	6/6	0.3899
Low	10^1.3^	36.7	0.57	6/6	10^1.4^	36.3	0.43	6/6	0.2228

*SD* Standard deviation, *Ct* Cycle threshold

^a^Viral Transport Medium- brain-heart infusion broth (pH 7.2), 10% (v/v) glycerol and antimicrobials per litre: 100 mg doxycycline, 100 mg enrofloxacin, 1000 mg penicillin-streptomycin, 5 mg Amphotericin B

^b^Egg Infectious Dose_50_ equivalents as determined by RT-rtPCR

^*^Statistically significant

### Effect of the transport medium on the isolation of H5N8 HPAI and H7N1 LPAI viruses from ostrich tracheal swabs

In the second experiment, virus isolation was performed on 10-swab pool samples spiked with either H5N8 HPAIV or H7N1 LPAIV. H5N8 HPAIV was readily isolated from ostrich tracheal swabs spiked with the highest concentration after just one passage and from all six eggs if stored in VTM, whereas when swabs were stored in PBS: glycerol only five eggs were positive on the first passage, with one sample requiring filtration at passage 2 due to bacterial growth (Table 3). When swabs were spiked with a medium virus concentrations dose, half of the eggs were positive after two or three passages without the need for filtration if stored in VTM, but if the swabs were stored in PBS: glycerol, only one of the eggs was positive after three passages, and the sample required filtration required at the third passage. Whereas all samples in both transport media spiked with the lowest virus concentration were still positive on RT-rtPCR with mean Ct values of 34.4 and 34.1 for VTM and PBS: glycerol respectively, H5N8 viruses could only be isolated from one egg each in the VTM- or PBS-stored swabs after three passages. VTM-stored swabs had slightly higher H5N8 virus titres with correspondingly lower Ct values than those stored in PBS: glycerol of 10^6.7^ vs. 10^6.6^ EID_50_/ ml for the high dose and 10^4.5^ vs. 10^4.4^ EID_50_/ ml for the medium dose. The single egg isolate at the lowest virus concentration where the titre of the recovered virus was however slightly higher in the PBS: glycerol medium with a titre of 10^3.1^ compared to 10^3.0^ EID_50_/ ml. Although not statistically significant (*p* value, = 0.505) overall, IAV was more frequently isolated (10/ 18) when swabs were stored in VTM than in PBS: glycerol (7/ 18).

**Table 3:** Comparison of H5N8 HPAI virus isolation efficiency from tracheal swabs (10/ pool) stored in different transport media

Media	Spiking dose	Virus isolation results (number of passages performed)						Total Positive	Allantoic fluid mean virus titre in EID_**50**_ equivalents^b^/ ml (mean Ct value)
		Replicate 1	Replicate 2	Replicate 3	Replicate 4	Replicate 5	Replicate 6		
VTM	High	**+** (1)	**+** (1)	**+** (1)	**+** (1)	**+** (1)	**+** (1)	6/6	10^6.7^ (22.4)
	Medium	**+** (2)	- (3)	**+** (2)	- (3)	**+** (3)	- (3)	3/6	10^4.5^ (29.4)
	Low	- (3/2^†^)	- (3)	**+** (3)	- (3)	- (3/2^†^)	- (3/2^†^)	1/6	10^3.0^ (34.4)
PBS: glycerol	High	**+** (1)	**+** (1)	- (2^†^)	**+** (1)	**+** (3)	**+** (1)	5/6	10^6.6^ (22.8)
	Medium	**+** (3/3^a^)	- (3)	- (3)	- (3)	- (3/2^a^)	- (3)	1/6	10^4.4^ (30.0)
	Low	**+** (2)	- (3)	- (3 /2^a^)	- (3)	- (3)	- (3)	1/6	10^3.1^ (34.1)

*Ct* Cycle threshold

+ Positive

- Negative

^a^Passage number where the sample was filtered

^b^Egg Infectious Dose_50_ equivalents as determined by RT-rtPCR

H7N1 LPAIV was isolated from all six eggs samples spiked with highest viral concentration in the swabs stored in VTM as well as PBS: glycerol (Table 4), but for VTM-stored swabs 4/ 6 eggs were positive after just one passage without the need for filtration in any eggs, whereas PBS: glycerol-stored swab fluids all required at least two passages, with filtration in two samples. No virus was isolated from swab pools spiked with medium and low virus concentrations of H7N1 LPAIV for VTM of PBS: glycerol medium. Notably more filtration was required to remove bacteria from PBS: glycerol-stored samples (six instances) than those in VTM (none), and higher virus titres of 10^5.5^ vs. 10^5.1^, 10^3.4^ vs. 10^2.9^ and 10^1.4^ vs. 10^1.3^ EID_50_/ ml were recovered from VTM-stored swabs compared to PBS: glycerol for high, medium and low spiking doses, respectively.

**Table 4:** Comparison of H7N1 LPAI virus isolation efficiency from tracheal swabs (10/ pool) stored in different transport media

Media	Spiking dose	Virus isolation results (number of passages performed)						Total Positive	Allantoic fluid mean virus titre in EID_**50**_ equivalents^b^/ ml (mean Ct value)
		1	2	3	4	5	6		
VTM	High	**+** (1)	**+** (1)	**+** (1)	**+** (1)	**+** (2)	**+** (2)	6/6	10^5.5^ (23.1)
	Medium	- (3)	- (3)	- (3)	- (3)	- (3)	- (3)	0/6	10^3.4^ (29.9)
	Low	- (3)	- (3)	- (3)	- (3)	- (3)	- (3)	0/6	10^1.4^ (36.3)
PBS: glycerol	High	**+** (2)	**+** (2)	**+** (2/1^a^)	**+** (2)	**+** (2/1^a^)	**+** (2)	6/6	10^5.1^ (24.2)
	Medium	- (3)	- (3)	- (3)	- (3/1^a^)	- (3)	- (3/1^a^)	0/6	10^2.9^ (31.4)
	Low	- (3)	- (3)	- (3/1^a^)	- (3)	- (3/1^a^)	- (3)	0/6	10^1.3^ (36.7)

*Ct* Cycle threshold

+ Positive

- Negative

^a^Passage number where the sample was filtered

^b^Egg Infectious Dose _50_ equivalents as determined by RT-rtPCR

## Discussion

To maintain the country’s international status as the largest export producer of ostrich products, South African farmers must adhere to strict bio-security requirements and undertake rigorous and costly testing to prove freedom of IAV infection. Pooling 5 ostrich tracheal swabs is the current standard practice as pooling increases not only testing throughput, but it also reduces the number of tests needed to meet sample size requirements and consequently the test costs [9, 13]. Pools of up to 11 swabs for chicken or turkey swabs are validated and used internationally [7, 9, 11, 13, 14], therefore in the current study we investigated the effects of also increasing ostrich tracheal swab pools to 10. Our results showed that increasing the ostrich swabs to 10 per pool had no detrimental effect on the sensitivity of the RT-rtPCR assay in detecting H5N8 HPAIV or H7N1 LPAIV; and doubling the swab pool size even seemed to improve the sensitivity of IAV detection. We contemplated that this phenomenon was possibly caused by the concentrating effect of the larger number of swabs pooled into the same 3 ml volume of transport medium: some virus particles trapped in the viscous ostrich mucous or other cellular material could be prevented from being drawn deeper into the swabs, concentrating them slightly in the lower volume remaining for RNA extraction.

LPAIV infections are more difficult to detect than HPAIV because levels of genome in swabs are generally lower than are typically found in HP infections [9] but this hasn’t yet been experimentally determined for ostriches. In other studies, at low-level LPAIV infection prevalence, testing pools of 11 detected slightly more infections while at higher prevalence, testing pools of 5 or 6 performed better [13]. If LPAIV does circulate at lower levels in ostriches, it is likely that swab pools of 10 will be sufficient to detect HPAI virus as well.

Identifying the IAV subtypes detected during surveillance of ostriches is of critical importance, not only to rule out or confirm the presence of a notifiable subtype, but also to resolve “continuing” outbreaks diagnosed on suspect serology [15]. Even though next generation whole genome sequencing performed directly on clinical samples has been instrumental in identifying pathogens [4], the OIE still regards virus isolation as the gold standard for agent identification [7], and the antigens themselves are valuable diagnostic reagents. Despite low Ct values, the success rate of IAV isolation from ostrich tracheal swabs has been poor, and this is of serious concern to national and international regulators alike. Numerous studies have extensively evaluated the factors in sample collection and transport that maximise the chances of IAV detection and isolation such as swab type, transport medium, length of time between collection and testing and prolonged storage conditions [10, 16]. Flocked swabs are reportedly superior to other swab types in capturing virus particles during swabbing [10] but the standard practice in South Africa and elsewhere is to use rayon-tipped swabs. Rayon-tipped swabs are substantially cheaper (44 % based on local pricing) than flocked swabs yet slightly less effective than flocked swabs in recovering IAVs [10], however the efficiency loss of flocked vs non-flocked swabs is negligible when swabs remain immersed overnight in an appropriate transport medium and if they are vigorously vortexed prior to processing [16].

The transport medium is another factor that has been widely investigated. PBS as a transport medium was demonstrated to significantly reduce the sensitivity of IAV isolation compared to a protein-buffered media [10]. The standardized use of PBS: glycerol as a transport medium could therefore also be a contributing factor to the historically low isolation rate of IAVs from ostrich tracheal swabs, but in South Africa, despite OIE recommendations [7] and primarily due to cost, antimicrobials are not added to the transport medium. Ostrich tracheal mucus and saliva on swabs are rich sources of proteins, enzymes, carbohydrates and electrolytes in the standard PBS: glycerol transport medium, which, combined with any break in the cold chain, provides ample opportunity for bacterial or fungal growth in ostrich tracheal swabs [12]. Even though the standard operating procedure requires swab pools to be treated with antimicrobials prior to inoculation into ECEs [7], few IAVs are isolated from ostrich tracheal swabs, even if the Ct value of the RNA titre is low, indicating the presence of virus in the sample. In a recent study we discovered that microbes found in the ostrich trachea propagate in the standard PBS: glycerol medium after sampling and directly affect viral viability. Ostrich tracheal swab pools submitted to a national facility for routine screening were cultured and we identified 13 bacteria, 1 yeast, and 2 fungal species. *Streptococcus* sp., *Pantoea* sp., and *Citrobacter freundii* produced extracellular metabolites that caused substantial reductions in IAV titers of 99.99 to 99.97 %, whereas *Streptomyces*, *Corynebacterium*, *Staphylococcus*, *Arthrobacter gandavensis*, *Pseudomonas putida*, and *Acinetobacter* spp. reduced the viability of IAV from 77.6 to 24.1 %. Although the identities of the bacterial proteins and their mechanism of viral inactivation were not determined, the disruption of the IAV envelope for example would render the virus non-viable, although the viral RNA might still be intact [12]. The results were supported in the present study where we demonstrated that whereas both H5N8 HPAIV and H7N1 LPAIV were detected by RT-rtPCR in all samples, upon isolation more eggs were positive when swabs were stored in VTM than in PBS: glycerol, overall higher virus titres were recovered, and fewer passages were required and less filtration for both H5N8 and H7N1 viruses. Filtration ultimately removes bacterial and fungal contamination, but it can also remove viable virus and reduces the sample volume considerable and should thus be avoided if possible [16]. The bacteria we previously identified in the ostrich tracheal swabs were all susceptible to erythromycin (15 μg) and sulphonamide (300 μg) [12] and their addition in the VTM would consequently reduce the need for filtration.

## Conclusion

Increasing ostrich tracheal swab pools from 5 to 10 swabs in 3 mls of transport medium had no detrimental effect on the sensitivity of the RT-rtPCR assay in detecting HPAI or LPAI viruses and can both improve the feasibility and increase sampling numbers for additional statistical confidence, and HPAI virus could be isolated from a greater proportion of swabs stored in VTM compared to PBS: glycerol without antibiotics.

## Methods

### Preparation of ostrich tracheal swab pools

The large size of ostriches and ethical considerations precluded housing enough of them under the high containment conditions required for experimental infections to produce the numbers of tracheal swabs required from infected and non-infected birds. Therefore, we conducted spiking experiments of ostrich tracheal swabs with egg cultured IAVs. Eight hundred freshly slaughtered ostrich carcases, from a farm that tested negative for the presence of IAV during routine pre-movement testing (unpublished laboratory results), were sampled at the abattoir in Oudtshoorn, Western Cape Province, in March 2020. Tracheal swabs were collected with sterile rayon tipped swabs (COPAN Diagnostics Inc.) and shipped on ice packs to the Western Cape Provincial Veterinary Laboratory within 24 h.

To prepare the live viruses for spiking, egg alantoic fluids containing LPAI strain A/Ostrich/South Africa/OUD/2012 (H7N1) or HPAI strain A/Speckled pigeon/South Africa/08-004B/2017(H5N8) at egg infectious doses (EID) of 10^7.7^ EID_50_/ ml and 10^8.5^ EID_50_/ ml, respectively were titrated at 10-fold and 2-fold serial dilutions in sterile nuclease-free water and tested in triplicate using quantitative RT-rtPCR as described below, to determine the high (10^− 1^ dilution), medium (10^− 3^ dilution), and low (10^–4.6^ dilution) virus concentrations that would yield approximate cycle threshold (Ct) values in the regions of 20–25, 26–30 and 31–35 respectively.

To prepare the tracheal swab pools, either nine or four tracheal swab tips were placed into sterile 5 ml tubes (20 mm diameter) containing 3 ml of viral transport medium (VTM) or 50 % (v/v) PBS: glycerol (pH 7.2) without antimicrobials. VTM consisted of brain-heart infusion (BHI) broth (pH 7.2) (Oxoid Ltd), 10% (v/v) glycerol and the following antimicrobials per litre: 100 mg doxycycline (Mylan), 100 mg enrofloxacin (Cipla), 1000 mg penicillin-streptomycin (Sigma-Aldrich, Merck), and 5 mg Amphotericin B (Bristol-Myers Squibb).

Sterile rayon-tipped swabs tips were placed into each H5N8 HPAIV or H7N1 LPAIV low, medium, or high concentration suspension, vortexed briefly and incubated at room temperature for 2 minutes to allow each tip to absorb the fluid. A single tip was successively removed from each virus suspension and placed into the prepared tubes containing either four or nine non-spiked tracheal swabs to prepare six replicates each of five- and ten-tracheal swab pools in VTM or PBS: glycerol. The pooled samples were refrigerated at 4 to 8 °C for 48 h before testing, as surveillance samples from ostriches typically reach a test laboratory 2 to 4 days after sampling in the field.

### Nucleic acid extraction and quantitative RT-rtPCR

Total nucleic acids were extracted from 200 μl of the alantoic fluids or swab fluids with the IndiSpin QIAcube HT Pathogen Kit (Indical Biosciences) and QIAcube HT system (QIAGEN). Two microliters of a synthetic RNA internal positive control (IPC) VetMax™ Xeno™ RNA (10,000 copies/μl) (Applied Biosystems Thermo Fisher Scientific) was included in nucleic acid extraction to detect PCR inhibition. VetMAX™-Gold AIV detection kits (Applied Biosystems Thermo Fisher Scientific) were used for quantitative RT-rtPCR targeting the matrix and the nucleoprotein genes of IAV and the Xeno™ RNA IPC target. Four microliters of the total nucleic acid eluate and 8.5 μl of master mix were used per reaction. The LightCycler® 480 Instrument II (Roche Molecular Diagnostics) was used for the quantitative RT-rtPCR reaction with the recommended thermal cycle profile. Samples with a cycle threshold (Ct) value of ≤ 36.9 were interpreted as positive, samples with a Ct value of 37 to 39 were considered to be weak positive, and samples with Ct values greater than 39 or where no Ct value was recorded were considered to be negative. Standard curves for the two viruses were generated from 10-fold serial dilutions of the alantoic fluids prior to extractions, with each dilution was tested in triplicate. Standard linear regression formulae of y = − 3.5425x + 44.173 and y = − 3.2779x + 40.975 were calculated by the software for the H5N8 HPAI and H7N1 LPAI viruses respectively.

### Virus isolation

Prepared tracheal swab pools (10 swabs/ pool only) were shipped on ice packs to NOSA Testing (Pty) Ltd. in Pretoria where they were stored at − 80 °C until testing and were only defrosted just prior to testing. Virus isolation in 9–11-day old SPF ECEs (AviFarms, (Pty Ltd), Pretoria) was performed in a BLS-2+ laboratory and according to the international standard procedure [7], but using six eggs per sample per passage. A positive result was determined by embryo morphology, haemagglutination (HA) tests with chicken red blood cells and neutralisation tests as prescribed. Where bacterial and/or fungal contamination was present, inoculum was passed through 0.8/0.2 μm filters (Pall Corporation, Acrodisc Syringe Filters, Separations) before subsequent passages were performed. At least three passages were performed on samples that were reported as negative and quantitative RT-rtPCR was performed on each egg’s alantoic fluid.

### Statistical analysis

The significance of the mean RT-rtPCR Ct values obtained for five- and ten-swab pools in VTM or PBS: glycerol was tested using a two-tailed paired t-test, where a *p* value of ≤ 0.05 was considered significant. Fisher’s Exact Test was used to calculate the significance (p value ≤0.05) of the proportion of samples where virus was isolated between the group that contained standard 50 % v/v PBS glycerol transport medium and groups that contained VTM. Calculations were performed using Microsoft Excel software.

## Data Availability

All data generated or analysed during this study are included in this published article.

## Abbreviations

EU: European Union
DAFF: Department of Agriculture Forestry and Fisheries
IAV: Influenza A virus
LPAI: Low pathogenicity avian influenza
LPAIV: Low pathogenicity influenza A virus
HPAI: High pathogenicity avian influenza
HPAIV: High pathogenicity influenza A virus
OIE: *Office International des Epizooties;* World Animal Health Organization
RT-rtPCR: Reverse transcription real time polymerase chain reaction
PBS: Phosphate buffered saline
SPF: Specific pathogen free
ECE: Embryonated chicken eggs
VTM: Virus transport medium
EID: Egg infectious dose
IPC: Internal positive control
